# First-Principles Insights into Coverage-Dependent Water Adsorption Mechanisms on Representative Lunar Regolith Mineral Surfaces

**DOI:** 10.3390/ma19173805

**Published:** 2026-09-07

**Authors:** Xinnan Deng, Yue Hong, Xueli Wang, Xiuming Ye, Hongtao Xue, Chengdan He, Jin Wang, Fuling Tang

**Affiliations:** 1 School of Materials Science and Engineering, Lanzhou University of Technology, Lanzhou 730050, China; 13308341370@163.com (X.D.); 13519747825@163.com (Y.H.); xueht@lut.edu.cn (H.X.); 2 State Key Laboratory of Advanced Processing and Recycling of Nonferrous Metals, Lanzhou University of Technology, Lanzhou 730050, China; 3 National Key Laboratory on Vacuum Technology and Physics, Lanzhou Institute of Physics, Lanzhou 730000, China

**Keywords:** first-principles calculations, lunar regolith minerals, water adsorption, surface coverage, hydrogen bonding, HSAB principle

## Abstract

Water retention on the lunar surface is governed by water–mineral interactions, yet the atomic-scale transition from isolated adsorption to high-coverage water accumulation remains insufficiently understood. We perform spin-polarized first-principles calculations to investigate single- and multi-water adsorption on representative surfaces of four major lunar regolith minerals: CaAl_2_Si_2_O_8_, MgFeSi_2_O_6_, FeTiO_3_, and Mg_3_FeSi_2_O_8_. Single-water adsorption reveals that H_2_O preferentially anchors at exposed metal sites via O-M coordination, with Ti and Fe sites exhibiting stronger initial binding than Mg, Ca, or Al sites. The Hard–Soft Acid–Base (HSAB) principle provides a qualitative framework for this low-coverage site preference based on Lewis acidity. Specifically, the accessible d-orbitals and localized states of Ti/Fe centers introduce substantial covalent orbital coupling and interfacial polarization, which effectively reinforce the binding with the hard O-donor of water. However, as water coverage increases, the stabilization mechanism undergoes a fundamental transition. At low coverage, adsorption is localized and site-specific, governed by cation acidity. At high coverage, the formation of laterally connected hydrogen-bonded networks becomes the dominant stabilizing factor, and the overall adsorption behavior is increasingly dictated by surface topology and geometric compatibility for hydrogen-bond connectivity rather than by isolated cation acidity. This coverage-dependent evolution from electronic-driven anchoring to topology-driven network formation establishes a dual-stage cooperative mechanism for water accumulation on lunar mineral surfaces. Our findings suggest that models for volatile retention on airless bodies must account for both the electronic activity of surface cations and the structural topology of mineral surfaces.

## 1. Introduction

Water on the Moon is a central topic in lunar science because it connects volatile evolution, surface space weathering, polar volatile trapping, and future in situ resource utilization (ISRU). Lunar water could provide critical resources for life support, oxygen production, and propellant generation during sustained lunar exploration [1,2]. The lunar surface also offers a natural environment for understanding how volatiles are produced, transported, retained, and released on airless silicate bodies exposed to solar wind irradiation, micrometeoroid bombardment, and extreme thermal cycling [3]. Although the Moon was traditionally regarded as volatile-depleted, analyses of lunar volcanic glasses, apatite, and melt inclusions have demonstrated that water or hydroxyl can be preserved in lunar interior materials [4,5,6], indicating that lunar water may occur in multiple reservoirs and chemical states.

Orbital, impact, and exospheric observations have substantially advanced our understanding of lunar surface hydration. Early theoretical studies proposed that volatiles could migrate across the lunar surface and become trapped in permanently shadowed polar regions [7,8], and Lunar Prospector neutron spectrometer data later revealed enhanced hydrogen deposits near the lunar poles [9]. The Moon Mineralogy Mapper (M3) on Chandrayaan-1 detected widespread absorption features near 3 micrometers that are generally attributed to OH and/or H_2_O-bearing species [10,11]. Similar hydration signatures were reported from Cassini/VIMS and Deep Impact observations [12,13], while subsequent M3-based analyses showed that lunar OH/H_2_O abundance is controlled by latitude, local time, composition, temperature, and illumination conditions [14,15]. Additional evidence from SOFIA, LCROSS, Diviner, and polar M3 observations further confirmed the coexistence of molecular water, polar volatile enrichment, cold-trap stability, and surface-exposed water ice on the Moon [16,17,18,19,20]. LADEE measurements indicated that meteoroid impacts can liberate water from lunar soil [21], and solar-wind interaction and volatile migration models suggest that water-related species may be redistributed across the lunar surface before being trapped or released [22,23]. These findings indicate that lunar water is part of a dynamic system involving implantation, migration, trapping, release, and mineral surface retention.

Recent Chang’E-5 studies provide direct constraints on the mineralogical occurrence and retention of lunar surficial water. In situ spectral measurements detected water signals in lunar regolith and rock at the landing site [24], while returned-sample analyses showed that solar wind-derived OH/H_2_O is preferentially enriched on the outermost surfaces of olivine, plagioclase, and pyroxene grains [25]. High abundances of solar wind-derived hydrogen were also observed in Chang’E-5 lunar soil grain rims [26], and impact glass beads were proposed as an important solar wind-derived water reservoir that may contribute to the lunar surface water cycle [27]. Recent work further suggested that ilmenite may participate in surface water cycling in the Procellarum KREEP Terrane [28]. These findings highlight that lunar water retention is strongly linked to mineral surfaces, grain rims, and regolith microstructures.

The lunar regolith is mainly composed of silicate and oxide minerals, including pyroxene, plagioclase feldspar, olivine, and ilmenite, with mineral proportions varying between mare and highland regions [29,30]. These minerals expose chemically distinct metal cations, such as Fe, Ti, Mg, Ca, and Al, which may serve as anchoring sites for water molecules. At the atomic scale, the initial capture of H_2_O is expected to depend on the coordination environment, Lewis acidity, and electronic structure of exposed surface metal sites, whereas water accumulation at higher coverage may be increasingly affected by intermolecular hydrogen bonding. Therefore, a systematic comparison of water adsorption on representative lunar mineral surfaces is essential for clarifying mineral-dependent water retention.

Previous mineral-water interface studies have provided important insights into water adsorption on silicate and oxide surfaces. First-principles and atomistic simulations of forsterite surfaces showed that water adsorption depends strongly on surface orientation, exposed metal sites, and surface stability [31,32]. DFT calculations on olivine (010) surfaces demonstrated that water adsorption involves metal-water coordination and hydrogen bonding with surface oxygen atoms, and that cation substitution can modify adsorption strength [33]. Experimental and theoretical studies on microcline feldspar (001) revealed that surface hydroxyl groups and the arrangement of the first adsorbed water molecules influence subsequent water binding [34]. DFT studies on calcium aluminosilicate surfaces also showed that water adsorption is sensitive to local surface composition and the coordination environment of Ca, Al, and Si sites [35]. In parallel, solar wind implantation experiments and simulations have demonstrated that implanted hydrogen can interact with oxygen in silicate materials to form hydroxyl- or water-related species [36,37,38], while thermal desorption studies of lunar analogs and Apollo regolith indicate that water binding and release depend on coverage, composition, and temperature [39,40]. However, most existing studies focus on individual minerals, specific surfaces, or isolated adsorption states, including our previous single H_2_O molecule adsorption research [41].

Despite these advances, a unified first-principles comparison of single- and multi-water adsorption on major lunar mineral surfaces remains limited. In particular, the preferred initial anchoring sites of isolated water molecules need to be identified under the same computational framework, and the evolution from isolated adsorption to higher water coverage remains insufficiently understood. Since lunar surface hydration depends not only on the strongest adsorption site but also on the ability of mineral surfaces to accommodate additional water molecules, a coverage-dependent adsorption study is required.

In this work, spin-polarized first-principles calculations were performed to investigate single- and multi-water adsorption on representative surfaces of four major lunar soil minerals: ilmenite FeTiO_3_, Fe-bearing olivine Mg_3_FeSi_2_O_8_, anorthite CaAl_2_Si_2_O_8_, and Fe-bearing pyroxene MgFeSi_2_O_6_. Surface energy calculations were used to evaluate the stability of different low-index surfaces. Single-water adsorption calculations were then performed to identify preferential anchoring sites and representative adsorption surfaces. Electronic structure analyses, including ELF, PDOS, charge-density difference, Bader charge, and COHP, were conducted to reveal the bonding origin of site-dependent adsorption. Finally, coverage-dependent adsorption models at approximately 10%, 25%, 50%, and 100% water coverage were constructed to clarify the transition from isolated water adsorption to hydrogen-bonded water networks. This study provides atomic-scale insight into the mineral-dependent capture and accumulation of water molecules on lunar soil mineral surfaces. We further note that our high-coverage models (≥75%) are based on ideal flat surfaces; real surface irregularities (steps, pits, convex/concave features) would perturb dense water layers, and thus these results represent an upper-bound estimate for the most regular surface topologies. We also note that the present work focuses on ideal crystalline surfaces, whereas the real lunar regolith is subjected to space weathering—including solar wind ion irradiation, cosmic ray bombardment, and micrometeorite impacts—which creates amorphous rims, glass aggregates, and localized radioactive anomalies (e.g., U and Th enrichment in KREEP terranes). These effects are beyond the scope of the current study but represent important directions for future investigations.

## 2. Methodology

### 2.1. Computation Method

In this study, all first-principles calculations were performed within the framework of spin-polarized density functional theory (DFT) using the Vienna Ab initio Simulation Package (VASP) [42]. The electron-ion interactions were described using the projector augmented wave (PAW) method, and the exchange-correlation interactions were treated by the generalized gradient approximation (GGA) with the Perdew-Burke-Ernzerhof (PBE) functional [43]. To account for long-range dispersion interactions between water molecules and mineral surfaces, the Grimme DFT-D3 correction was employed throughout the calculations [44]. For transition-metal-containing systems, the DFT+*U* method was adopted to describe the localized d electrons, with effective *U* values of 4.0 eV for Fe and 3.0 eV for Ti [45,46]. The plane-wave cutoff energy was set to 550 eV. The convergence criterion for the total energy was set to 1.0 × 10^−7^ eV, and the maximum residual force on each atom was less than 0.05 eV Å^−1^. Spin-polarized calculations were performed in all simulations. The Brillouin zone was sampled using Monkhorst–Pack *k*-point meshes, and the k-point grids were selected according to the size and symmetry of different slab models [47]. For electronic structure calculations, denser *k*-point meshes were used to obtain smooth and reliable density of states. A vacuum layer of 20 Å was introduced along the surface-normal direction to avoid artificial interactions between periodic images.

The adsorption energy of a single water molecule was calculated as follows:

(1) $$E_{ads}=E_{slab+H_{2}O}-E_{slab}-E_{H_{2}O}$$

where $E_{slab+H_{2}O}$, $E_{slab}$, and $E_{H_{2}O}$ are the total energies of the water-adsorbed slab, the clean slab, and the isolated water molecule, respectively. A more negative adsorption energy indicates a stronger and more stable adsorption configuration.

For multiple water adsorption, the average adsorption energy was defined following commonly used treatments for coverage-dependent adsorption systems as [47,48,49]:

(2) $$E_{avg}=\frac{E_{slab+nH_{2}O}-E_{slab}-nE_{H_{2}O}}{n}$$

where *n* is the number of adsorbed water molecules. To further evaluate the energetic contribution of an additionally adsorbed water molecule, the differential adsorption energy was calculated by [49,50]:

(3) $$E_{diff}=E_{slab+nH_{2}O}-E_{slab+\left(n-1\right)H_{2}O}-E_{H_{2}O}$$

The water coverage was defined as:

(4) $$\theta =\frac{n}{N_{site}}\times 100\%$$

where $N_{site}$ represents the number of available surface metal adsorption sites in the selected surface supercell. Based on this definition, adsorption models with coverages of approximately 10%, 25%, 50%, and 100% were constructed.

The average surface energy of the clean slab was calculated using the conventional slab-based surface energy expression [51,52]:

(5) $$\gamma_{avg}=\frac{E_{slab}-NE_{bulk}}{2A}$$

where $E_{slab}$ is the total energy of the relaxed clean slab, $E_{bulk}$ is the energy per formula unit of the corresponding bulk mineral, *N* is the number of formula units in the slab, and *A* is the surface area of one side of the slab. For slabs with inequivalent upper and lower terminations, the calculated value represents the average surface energy of the two exposed surfaces.

The electron localization function (ELF), projected density of states (PDOS), charge density difference, Bader charge analysis, and crystal orbital Hamilton population (COHP) were employed to analyze the electronic origin of water adsorption [52,53,54,55,56,57]. The charge density difference was calculated according to:

(6) $$∆\rho =\rho_{slab+H_{2}O}-\rho_{slab}-\rho_{H_{2}O}$$

where $\rho_{slab+H_{2}O}$, $\rho_{slab}$, $\rho_{H_{2}O}$ are the charge densities of the adsorbed system, the clean slab, and the isolated water molecule with the same atomic positions, respectively.

### 2.2. Computational Model

Four representative lunar soil minerals were selected, including ilmenite FeTiO_3_, Fe-bearing olivine Mg_3_FeSi_2_O_8_, anorthite CaAl_2_Si_2_O_8_, and Fe-bearing pyroxene MgFeSi_2_O_6_. The bulk structures were first optimized, and slab models were subsequently constructed from the optimized bulk phases following the general slab-supercell approach for surface calculations [51,52].

For single-water adsorption, one H_2_O molecule was placed near the exposed surface metal sites, including Fe, Ti, Mg, Ca, and Al, depending on the surface composition, because previous studies have shown that water adsorption on silicate and oxide surfaces is commonly governed by metal-water coordination and hydrogen bonding with surface oxygen atoms [33,35,36,37,47]. Different initial orientations were examined, and the most stable configuration was identified based on adsorption energy and optimized geometry.

For coverage-dependent adsorption, representative surfaces were selected according to surface stability and single-water adsorption strength. Multiple water molecules were then adsorbed to construct approximately 10%, 25%, 50%, and 100% coverage models. At low coverage, water molecules were preferentially placed at the most stable metal anchoring sites. At higher coverages, both water-mineral interactions and water–water hydrogen bonding were considered in constructing the initial adsorption configurations, consistent with the established understanding that water adsorption on solid surfaces is strongly affected by the competition between molecule-surface binding and intermolecular hydrogen bonding [47,48,49,58,59].

This modeling strategy enables the adsorption process to be analyzed in two steps: single-water adsorption was used to identify the initial anchoring sites and bonding mechanism, while multi-water adsorption was used to reveal the coverage-dependent evolution from isolated adsorption to hydrogen-bonded water networks.

The present calculations employ idealized periodic crystalline surfaces to isolate the intrinsic interaction between H_2_O and representative mineral surface sites. Such models provide a controlled atomistic framework for comparing adsorption energetics and local bonding characteristics, but they do not fully reproduce the structural heterogeneity of actual lunar regolith. Real regolith particles may contain surface roughness, defects, pores, amorphous domains, and locally curved environments, which can modify the accessibility and coordination environment of adsorption sites. Therefore, the adsorption energies and coverage-dependent configurations obtained here should be regarded as an atomistic baseline for pristine mineral surfaces rather than a direct quantitative description of water behavior on realistic regolith particles.

## 3. Results and Discussions

### 3.1. Surface Stability and Selection of Representative Adsorption Surfaces

**Figure 1:**
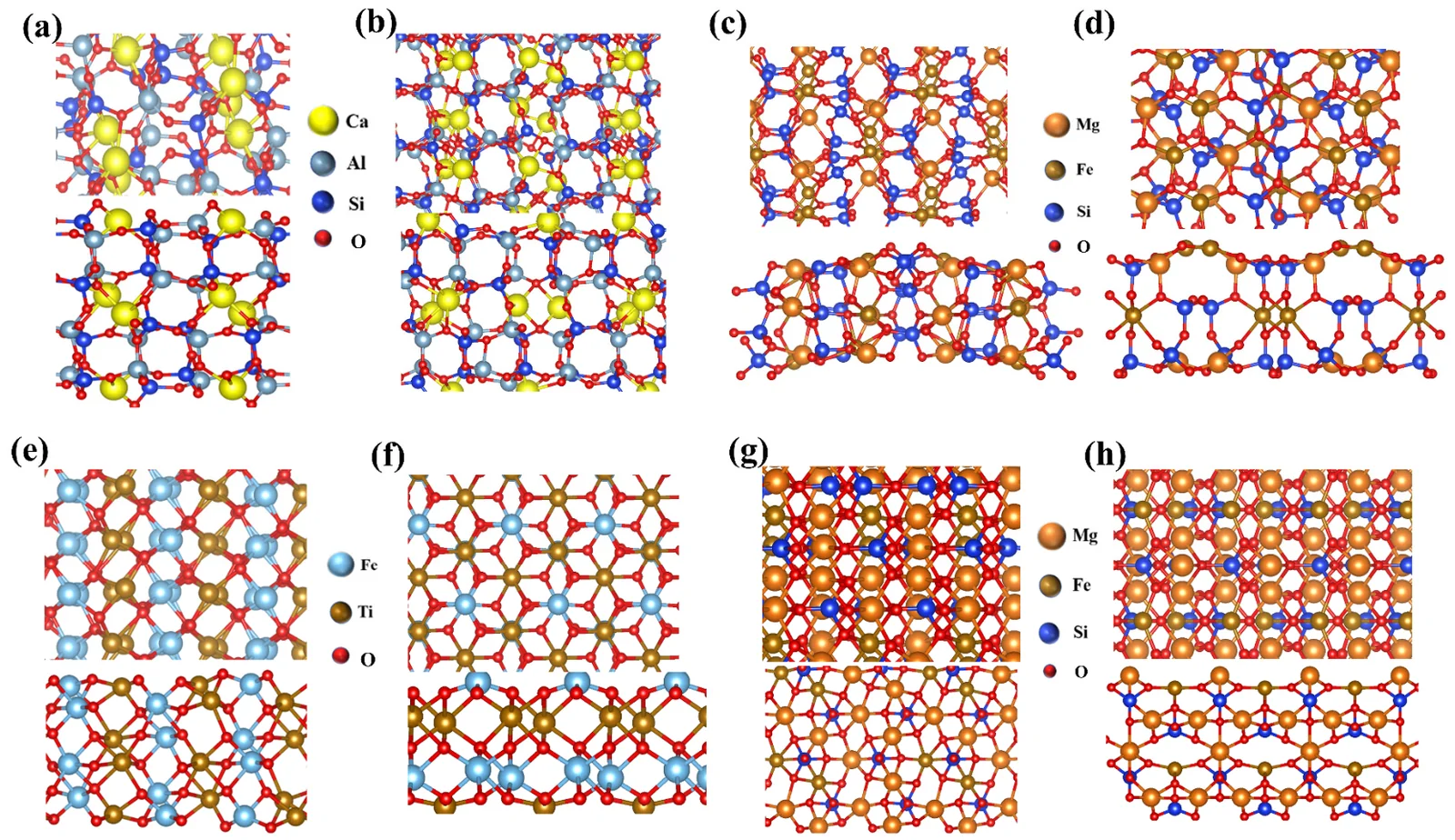
Optimized clean slab models of representative lunar mineral surfaces: (**a**) CaAl_2_Si_2_O_8_ (001), (**b**) (010); (**c**) MgFeSi_2_O_6_ (110), (**d**) (010); (**e**) FeTiO_3_ (100), (**f**) (001); (**g**) Mg_3_FeSi_2_O_8_ (010), and (**h**) (001).

To establish reliable surface models for investigating water adsorption on lunar minerals, two representative low-index surfaces were constructed for each mineral using the slab-supercell strategy commonly employed in first-principles surface calculations [51,52], including CaAl_2_Si_2_O_8_ (001)/(010), MgFeSi_2_O_6_ (110)/(010), FeTiO_3_ (100)/(001), and Mg_3_FeSi_2_O_8_ (010)/(001). The optimized clean slab models are shown in Figure 1. After structural relaxation, all slab models retained their primary crystal frameworks without significant surface reconstruction or collapse, indicating that the selected low-index surfaces are structurally stable and suitable for subsequent adsorption calculations.

Figure 1 shows that different surface terminations expose various metal cations, including Ca, Mg, Fe, Ti, and Al, which can serve as potential adsorption sites for H_2_O molecules. Since the initial interaction between water and mineral surfaces is primarily governed by coordination between the oxygen atom of H_2_O and exposed surface metal sites, both the thermodynamic stability of the clean surfaces and the adsorption activity of these exposed sites should be considered in the surface screening process.

The thermodynamic stability of the clean surfaces was further evaluated through surface energy calculations, and the results are summarized in Table 1. All investigated surfaces exhibit positive surface energies, indicating that the constructed slab models are thermodynamically feasible within the present computational framework. The calculated surface energies range from 0.280 to 1.287 J m^−2^, suggesting that all selected low-index surfaces can serve as reasonable adsorption substrates for water molecules.

As shown in Table 1, the differences in surface energy between some paired surfaces are relatively limited. For example, FeTiO_3_ (001) and (100) show surface energies of 0.542 and 0.574 J m^−2^, respectively, while CaAl_2_Si_2_O_8_ (001) and (010) exhibit values of 0.633 and 0.569 J m^−2^. These relatively close values indicate that both surfaces in each pair possess comparable thermodynamic stability. Under such conditions, the stronger single-water adsorption energy becomes a more direct criterion for evaluating the surface affinity toward water molecules.

In contrast, for Mg_3_FeSi_2_O_8_ and MgFeSi_2_O_6_, larger differences in surface energy are observed between the two considered surfaces. In both cases, the differences in surface energy reflect the distinct surface terminations and local coordination environments exposed by the two crystal orientations. The higher-energy surfaces may expose more coordinatively unsaturated metal sites, which can provide more favorable local environments for water adsorption.

It should be noted that surface energy mainly characterizes the thermodynamic stability of a clean mineral surface and does not directly determine its affinity toward water molecules. Therefore, in this section, surface energy was used only as a preliminary criterion to evaluate the structural reasonableness and thermodynamic feasibility of the constructed slab models. The positive surface energies and the absence of obvious structural collapse after relaxation indicate that these candidate surfaces are suitable for subsequent water adsorption calculations. The final selection of representative adsorption surfaces will be further determined by combining the surface stability with the single-water adsorption behavior discussed in the following section.

### 3.2. Single Water Adsorption: Identification of Initial Anchoring Sites

**Figure 2:**
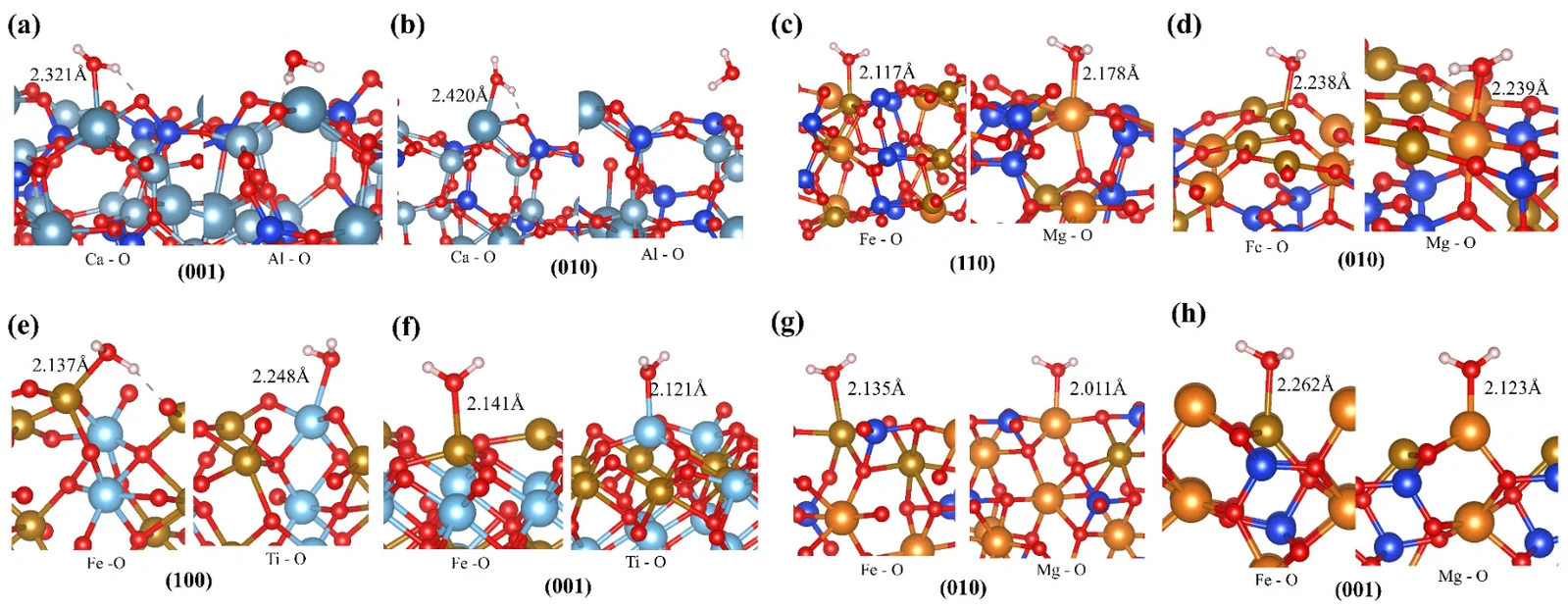
Optimized single-water adsorption configurations on different lunar mineral surfaces: (**a**,**b**) CaAl_2_Si_2_O_8_ (001) and (010); (**c**,**d**) MgFeSi_2_O_6_ (110) and (010); (**e**,**f**) FeTiO_3_ (100) and (001); (**g**,**h**) Mg_3_FeSi_2_O_8_ (010) and (001).

**Figure 3:**
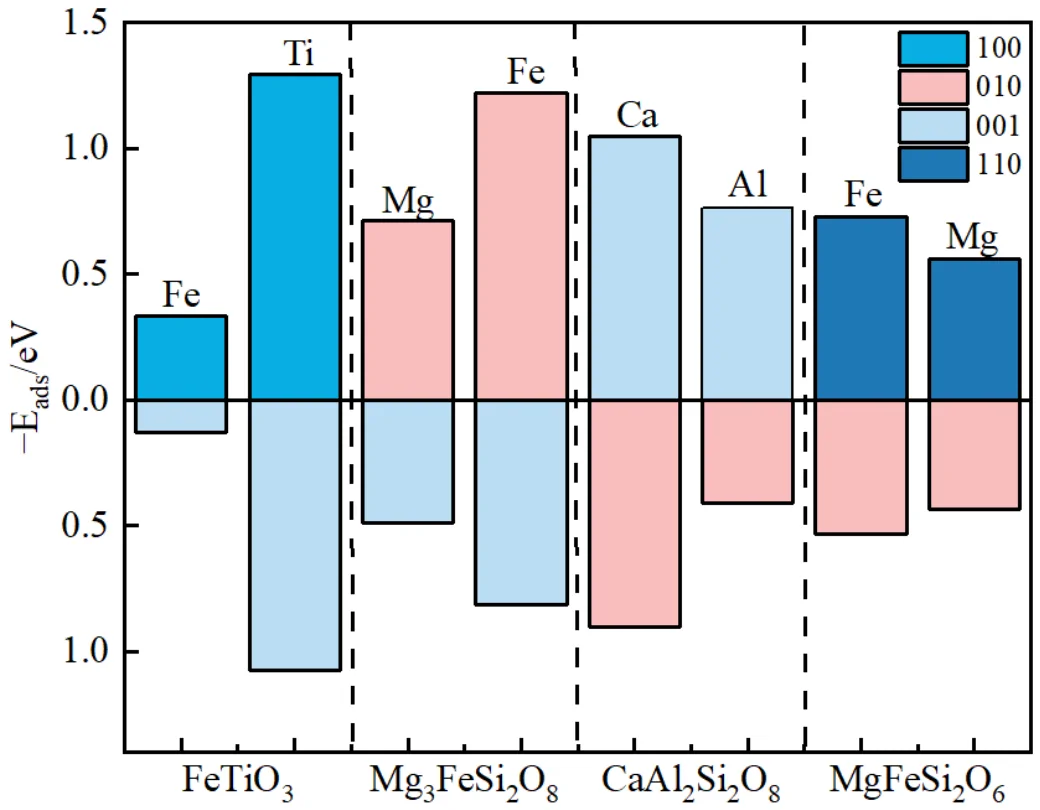
Comparison of single-water adsorption energies on different lunar mineral surfaces.

Single-water adsorption calculations were performed on all candidate surfaces to identify the preferential anchoring sites and to provide the structural basis for subsequent coverage-dependent adsorption. For each surface, H_2_O was initially placed near different exposed metal sites, including Fe, Ti, Mg, Ca, and Al sites, depending on the surface composition. The optimized adsorption configurations are shown in Figure 2, and the corresponding adsorption energies are compared in Figure 3.

As shown in Figure 2, all stable adsorption configurations exhibit molecular adsorption rather than dissociative adsorption. The O atom of H_2_O preferentially points toward an exposed surface metal cation, forming an O-M coordination interaction, while one or both H atoms are oriented toward neighboring surface oxygen atoms and may form auxiliary H···O hydrogen bonds. This adsorption motif indicates that the initial capture of H_2_O on lunar mineral surfaces is mainly governed by coordination between the lone-pair electrons of the water O atom and exposed surface metal cations, with neighboring surface oxygen atoms providing additional stabilization through hydrogen bonding. This adsorption motif is consistent with previous studies on water adsorption at silicate, feldspar, and calcium aluminosilicate surfaces [33,35,36,37,48].

The optimized M-O coordination distances provide further structural evidence for water-metal coordination. As marked in Figure 2, the M-O distances are mainly distributed within approximately 2.01–2.42 Å. This range suggests coordination-type adsorption rather than purely weak physisorption. However, the M-O distance alone does not completely determine the adsorption strength. For example, some Mg-O configurations show relatively short distances, but their adsorption energies remain weaker than those of Ti- or Fe-centered configurations. This indicates that the adsorption strength is controlled not only by geometric proximity, but also by the electronic activity, Lewis acidity, and local coordination environment of the surface metal site.

The adsorption energies in Figure 3 reveal a clear site-dependent adsorption trend. Transition-metal sites generally provide stronger water affinity than Mg- or Al-related sites. In FeTiO_3_, Ti sites exhibit much stronger adsorption than Fe sites on both investigated surfaces, indicating that Ti is the dominant water-anchoring center. For Fe-bearing silicate surfaces, Fe sites are more favorable than Mg sites, suggesting that O_w_-Fe coordination is more effective than O_w_-Mg coordination in stabilizing isolated water molecules. In contrast, Al-related configurations on CaAl_2_Si_2_O_8_ show weak direct coordination with H_2_O, while Ca sites act as the primary adsorption centers.

To rationalize the site-dependent adsorption trend, the Hard–Soft Acid–Base (HSAB) principle is introduced as a qualitative Lewis acid–base framework [60]. In the present systems, exposed surface metal cations can be regarded as Lewis acidic centers, whereas the oxygen atom of H_2_O (O_w_) acts as a hard O-donor Lewis base. Within this framework, water coordination is favored at sites that provide suitable Lewis acidity, coordinative unsaturation, and orbital accessibility. Therefore, HSAB is used here as a chemical descriptor for interpreting metal-site selectivity, while the quantitative adsorption strength is further determined by the local coordination geometry and electronic structure of each surface.

From this perspective, HSAB provides only a qualitative framework for describing the compatibility between the hard O-donor of H_2_O and exposed Lewis-acidic surface cations. The observed adsorption-energy differences cannot be attributed to HSAB matching alone. Instead, they arise from the combined effects of local coordination environment, surface accessibility, electronic polarization, orbital coupling, and structural relaxation. In particular, the stronger adsorption at Ti- and Fe-containing sites is accompanied by more pronounced O-2*p*–metal-*d* orbital interactions and larger interfacial charge redistribution, as further supported by the PDOS, differential charge-density, Bader-charge, and COHP analyses.

Therefore, the preferred initial anchoring sites can be summarized as Ti sites on FeTiO_3_, Fe sites on Mg_3_FeSi_2_O_8_ and MgFeSi_2_O_6_, and Ca sites on CaAl_2_Si_2_O_8_. This hierarchy reflects the combined effects of Lewis acidity, d-orbital participation, coordinative unsaturation, and surface accessibility, rather than a monotonic dependence on formal charge or M-O distance.

In addition to the metal-site effect, surface orientation also significantly affects the adsorption behavior. The same type of metal site can exhibit different adsorption strengths on different crystal surfaces because of variations in surface exposure, local coordination saturation, and spatial accessibility. Surfaces exposing more accessible or less saturated metal sites allow the water molecule to approach the surface more effectively and form a more stable O-M coordination structure. By contrast, when the metal site is partially shielded by the surrounding oxygen framework or embedded in a more compact surface environment, H_2_O tends to adopt a tilted geometry and the adsorption is weakened. This explains why the most stable adsorption surface does not necessarily correspond to the surface with the lowest surface energy, but rather to the surface that provides a more favorable local adsorption environment.

The combined comparison of Figure 2 and Figure 3 shows that stronger adsorption is generally associated with a more favorable coordination geometry and a more active metal center. Nevertheless, the relationship between M-O distance and adsorption energy is not strictly linear, because the adsorption process also involves water orientation, surface relaxation, and auxiliary hydrogen bonding with neighboring surface oxygen atoms. Therefore, the initial adsorption behavior should be understood as the result of coupled geometric and electronic effects, rather than a simple function of bond length or metal species alone.

Overall, the single-water adsorption results demonstrate that both the exposed metal species and the crystallographic surface orientation regulate the initial capture of water molecules on lunar mineral surfaces. Based on the adsorption configurations, M-O coordination distances, and adsorption energies, FeTiO_3_ (100), Mg_3_FeSi_2_O_8_ (010), CaAl_2_Si_2_O_8_ (001), and MgFeSi_2_O_6_ (110) were identified as the representative adsorption surfaces. These surfaces expose more favorable anchoring environments within their respective mineral systems and were therefore selected for the following electronic structure analysis and coverage-dependent multi-water adsorption calculations.

### 3.3. Brief Electronic Origin of Single-Water Adsorption on Mineral Surfaces

It is worth noting that the total adsorption energies on the Fe/Mg-bearing silicate surfaces, such as MgFeSi_2_O_6_ (110), consistently follow the trend of Fe > Mg, which matches the electronic bonding contributions evidenced by COHP and PDOS analyses. This indicates that the intrinsic electronic acidity and orbital coupling play a leading role in determining the initial single-water adsorption stability. Therefore, the higher chemical reactivity of transition-metal sites provides a useful initial guide for water anchoring on these mineral substrates.

This consistent behavior is further supported by the integrated COHP (ICOHP) values, where the total adsorption energy integrates both the intrinsic M-O bond strength and the energy cost associated with local surface relaxation upon water binding. On MgFeSi_2_O_6_ (110), although the Fe site induces a certain degree of local lattice strain during structural relaxation, the strong electronic coupling and orbital hybridization significantly overcome this geometric resistance. Consequently, the net adsorption energy at the Fe site remains more favorable than that at the Mg site, underscoring that the electronic factor dictates the initial stage of single-molecule capture, while geometric relaxation fine-tunes the exact energy differences.

Since the detailed electronic-structure analyses, including ELF, PDOS, and charge-density difference for single-water adsorption, have been systematically re-ported in our previous work [41], here we focus only on the key electronic features that are essential for understanding the coverage-dependent adsorption behavior. As established in Ref. [41], single-water adsorption on these mineral surfaces is characterized by molecular adsorption accompanied by local interfacial polarization rather than complete charge transfer. The PDOS results show that the Ti and Fe sites exhibit noticeable overlap between the O-2*p* states of H_2_O and the metal-*d* states in the valence region, indicating a certain degree of orbital hybridization and covalent contribution to the M-O coordination. In contrast, the Mg, Ca, and Al sites show relatively weak orbital hybridization, suggesting that their interactions with H_2_O are dominated by electrostatic or ionic contributions. The corresponding ELF and charge-density-difference results reported in Ref. [41] further indicate localized electron redistribution and interfacial polarization around the adsorption sites, rather than extensive electron transfer between H_2_O and the mineral surfaces. Importantly, the ELF distributions vary with the actual optimized adsorption geometries and surface cation sites. The O-centered localized electron density at Fe and Ti sites shows a more pronounced extension toward the surface, whereas the corresponding ELF features at Mg, Ca, and Al sites are relatively localized around the water O atom. These differences indicate that the spatial response of the O-centered electron density is site-dependent rather than identical for all adsorption configurations. The Bader charge analysis provides a quantitative measure of this adsorption-induced electronic redistribution [41]. Overall, the larger charge redistribution associated with the transition-metal sites, particularly Fe and Ti, is consistent with their stronger local polarization response and more pronounced orbital interactions with H_2_O. In the present work, this site-dependent electronic character is used as the basis for interpreting the subsequent coverage-dependent evolution, while the newly considered COHP results provide complementary information on the relative bonding characteristics of the corresponding M-O interactions.

However, it is important to note that these electronic differences primarily govern the initial capture of isolated water molecules. As will be shown in the following sections, with increasing water coverage, the overall adsorption stability becomes increasingly modulated by intermolecular hydrogen bonding and surface geometric constraints, which can partially compensate for or even override the intrinsic electronic preference of individual metal sites.

**Figure 4:**
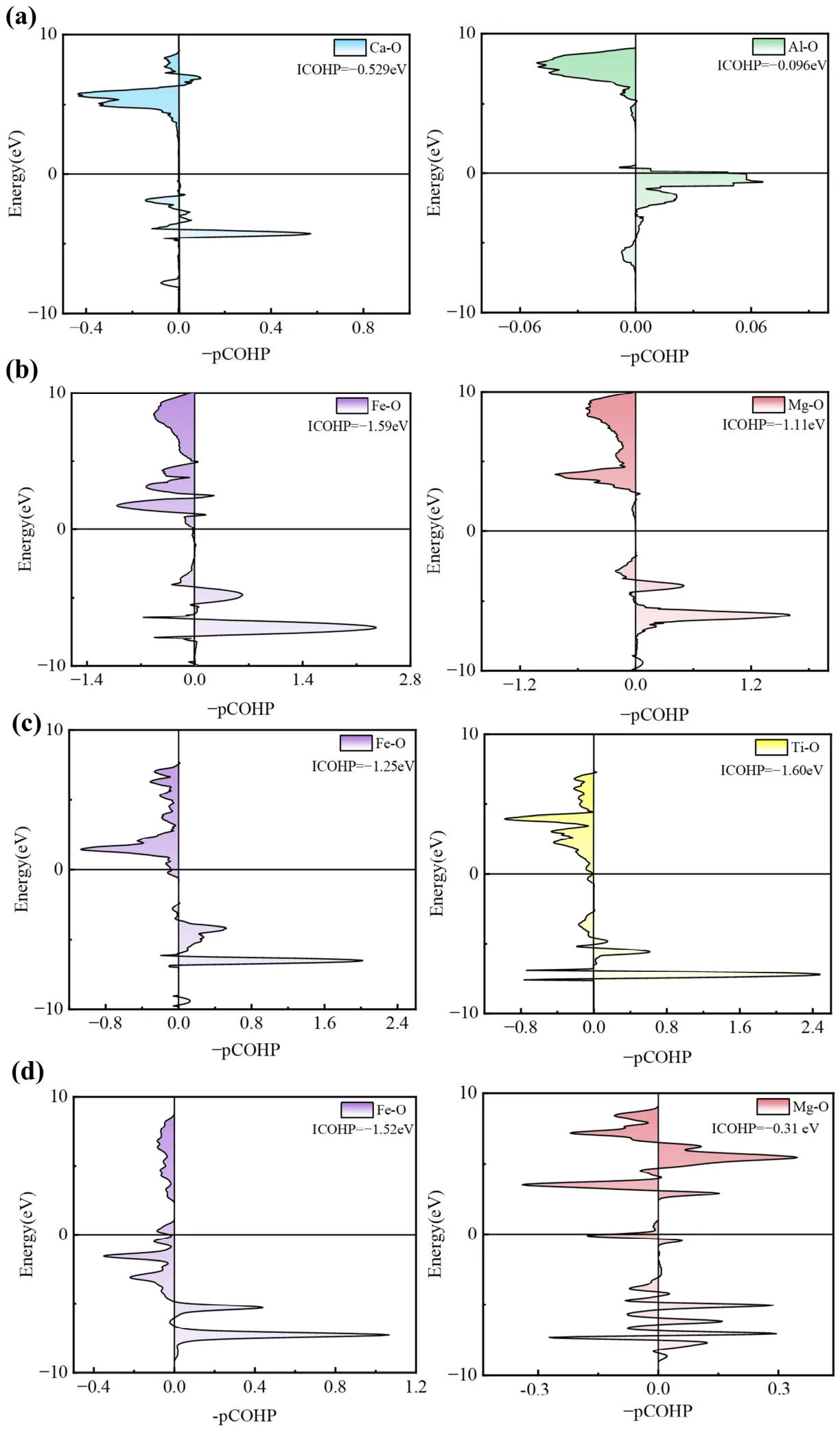
COHP analysis of M-O interactions between adsorbed H_2_O and surface metal sites. (**a**) CaAl_2_Si_2_O_8_ (001); (**b**) MgFeSi_2_O_6_ (110); (**c**) FeTiO_3_ (100); (**d**) Mg_3_FeSi_2_O_8_ (010).

The COHP analysis provides energy-resolved bonding/antibonding information (Figure 4), where positive -pCOHP and negative values denote bonding and antibonding contributions, respectively. The bonding characteristics of M-O interactions exhibit a clear site-dependent trend. Overall, transition-metal sites (Ti and Fe) display broader and stronger bonding peaks below the Fermi level, indicating efficient hybridization between water O-2*p* and metal *d*-orbitals over a wide energy range. For instance, the Ti-O bond on FeTiO_3_(100) (ICOHP = −1.60 eV) exhibits intense bonding occupations from approximately −7 to −3 eV, with significantly greater spectral width and intensity than the Fe-O bond (ICOHP = −1.25 eV), directly explaining the superior adsorption at Ti sites. Similarly, on Fe/Mg-bearing silicates, the ICOHP values of Fe-O bonds (−1.59~−0.99 eV) are systematically more negative than those of Mg-O bonds (−1.11~−0.31 eV), confirming the stronger covalent bonding capability of Fe sites. In contrast, main-group metal sites (Mg, Al, Ca) exhibit notably weaker -pCOHP features. The Mg-O and Al-O bonds show narrow and low-intensity bonding peaks, with non-bonding or weakly antibonding features appearing in certain energy regions, indicating that their *s*/*p*-*p* hybridization is far less efficient than the *d*-*p* coupling of transition metals. Notably, the Ca site (ICOHP = −0.53 eV), despite lacking *d*-orbitals, still exhibits stronger bonding occupations in the occupied region than the Al site (ICOHP = −0.10 eV), which is attributed to the larger ionic polarization and coordinative unsaturation of Ca. Furthermore, no simple monotonic relationship exists between bond length and adsorption strength (Table 2). For example, on Mg_3_FeSi_2_O_8_(010), the Mg-O distance (2.011 Å) is shorter than the Fe-O distance (2.135 Å), yet the Mg-O ICOHP (−0.31 eV) and adsorption energy (−0.71 eV) are substantially weaker than those of Fe-O (−0.99 eV and −1.22 eV). This indicates that adsorption stability is fundamentally dictated by electronic matching efficiency rather than mere geometric proximity.

Overall, the electronic structure results demonstrate that single-water adsorption on the selected lunar mineral surfaces is governed by a combined effect of metal-site coordination, local interfacial polarization, and weak hydrogen bonding. By framing this in the HSAB principle, we conclude that the initial water capture on lunar regolith is governed by Lewis acid–base coordination, where the accessible d-orbitals and stronger polarizability of Ti/Fe centers enable more effective orbital-mediated interfacial bonding compared with the more ionically interacting Mg, Ca, and Al sites.

### 3.4. Coverage-Dependent Adsorption of Multiple Water Molecules

**Figure 5:**
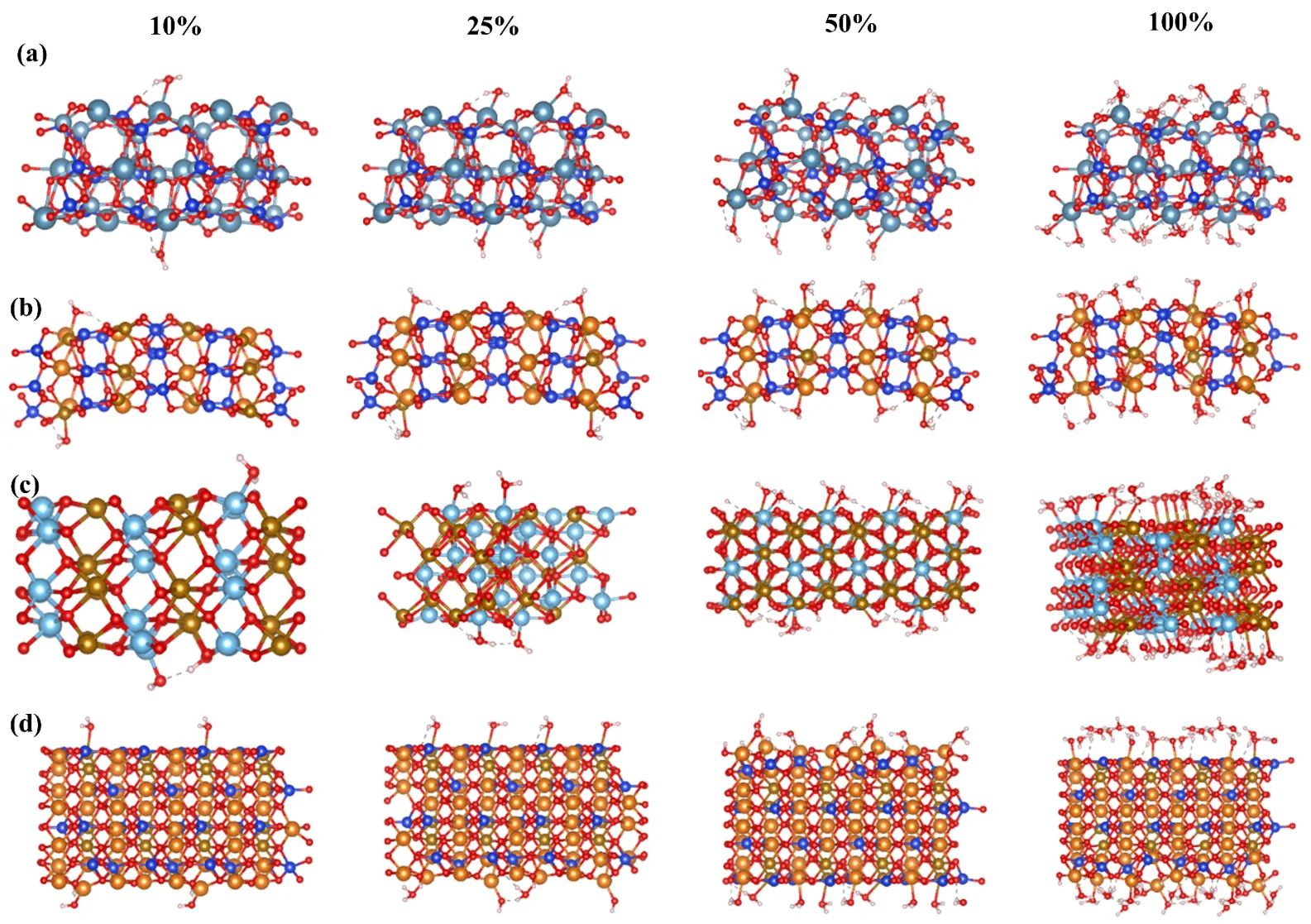
Optimized adsorption configurations of multiple H_2_O molecules on representative lunar mineral surfaces at different coverages: (**a**) CaAl_2_Si_2_O_8_ (001), (**b**) MgFeSi_2_O_6_ (110), (**c**) FeTiO_3_ (100), and (**d**) Mg_3_FeSi_2_O_8_ (010). For each surface, the configurations from left to right correspond to 10%, 25%, 50%, and 100% water coverage, respectively.

After identifying the preferential anchoring sites for isolated H_2_O molecules, coverage-dependent adsorption calculations were performed on the selected representative surfaces, including CaAl_2_Si_2_O_8_ (001), MgFeSi_2_O_6_ (110), FeTiO_3_ (100), and Mg_3_FeSi_2_O_8_ (010). Four water coverages, approximately 10%, 25%, 50%, and 100%, were considered to describe the adsorption evolution from isolated water molecules to high-coverage water accumulation. As shown in Figure 5, the adsorbed water molecules exhibit a clear coverage-dependent structural evolution. At 10% coverage, H_2_O molecules are spatially separated and mainly occupy the most favorable surface anchoring sites identified from the single-water adsorption calculations. In this stage, the adsorption configurations are dominated by direct O-M coordination between the water O atom and exposed surface metal cations, while the contribution from water-water interaction is limited because of the relatively large intermolecular distances. Therefore, the low-coverage adsorption behavior primarily reflects the intrinsic affinity of exposed metal sites toward isolated water molecules.

When the coverage increases to 25%, additional H_2_O molecules begin to occupy nearby surface regions, and the adsorbed molecules show more obvious orientation adjustment compared with the isolated adsorption state. Some H atoms are oriented toward neighboring surface oxygen atoms or adjacent water molecules, suggesting that local hydrogen-bond-assisted stabilization starts to participate in the adsorption process. However, most H_2_O molecules still remain close to the mineral surface, indicating that water-mineral interaction continues to play the dominant role at this coverage. This stage can therefore be regarded as a transition from independent site occupation to locally cooperative adsorption.

At 50% coverage, the adsorbed H_2_O molecules become more laterally connected on the mineral surfaces, indicating that intermolecular hydrogen bonding begins to play a more important role in stabilizing the adsorbed water layer. Such coverage-induced lateral organization of water molecules has been observed on several oxide and mineral-related surfaces, where local adsorption sites regulate the transition from isolated molecules to hydrogen-bonded assemblies [61,62,63]. Compared with the 10% and 25% configurations, water molecules are no longer only distributed around isolated metal centers; instead, they tend to extend along the surface and form local water clusters or short hydrogen-bonded chains. This structural evolution indicates that intermediate-coverage adsorption is controlled by two competing factors. On the one hand, the strongest anchoring sites are progressively occupied, and newly added H_2_O molecules must adsorb at relatively weaker sites or adopt less ideal orientations. On the other hand, the shorter distance between neighboring water molecules enables cooperative stabilization through water-water interactions. Therefore, the stability at intermediate coverage is determined by the balance between site saturation and local hydrogen-bond-assisted stabilization.

The transition from low to high coverage also reflects a gradual change in the dominant stabilization mechanism. At low coverage, the adsorption system is mainly governed by acid–base matching between isolated H_2_O molecules and the exposed metal sites. In this stage, Ti and Fe sites provide strong anchoring centers because their relatively soft or borderline Lewis acid character and active *d* orbitals favor stronger polarization-assisted O_w_-M coordination. As the coverage increases to 25–50%, these favorable anchoring sites become progressively occupied, and newly added H_2_O molecules must adsorb at less ideal surface positions or adopt tilted configurations. Under this condition, direct metal-water coordination is partly weakened, while hydrogen bonding among neighboring H_2_O molecules and between H_2_O and surface oxygen atoms becomes increasingly important.

At 100% coverage, the adsorbed water molecules form a quasi-continuous hydrogen-bonded network on the mineral surfaces, suggesting that water accumulation is increasingly stabilized by collective hydrogen-bond interactions rather than by independent adsorption at isolated metal sites [62,64]. The optimized high-coverage configurations are mainly characterized by lateral hydrogen-bond connectivity along the surface, although local vertical displacement of some water molecules is also observed. In this stage, part of the adsorbed water layer is stabilized by direct surface anchoring, while neighboring H_2_O molecules are further organized through intermolecular hydrogen-bond interactions. Therefore, the high-coverage adsorption state can be described as a surface-confined water layer characterized by lateral hydrogen-bond connectivity. This indicates that the dominant stabilization mechanism gradually changes from isolated metal-site anchoring to the cooperative interaction between the mineral surface and adsorbed water molecules.

As the coverage increases, the energetic contribution of individual surface sites becomes progressively coupled with intermolecular water–water interactions. At low coverage, the newly added H_2_O molecules preferentially occupy the most favorable exposed surface sites, resulting in relatively strong incremental stabilization. With increasing coverage, these favorable sites become progressively occupied, and the marginal adsorption energy becomes less negative, indicating reduced energetic gain for additional molecules. At higher coverage, the formation and extension of intermolecular H_2_O⋯H_2_O and H_2_O⋯surface-O hydrogen-bond contacts partially compensate for the reduced availability of strong metal anchoring sites. The resulting changes in *E_avg_* and *E_diff_* therefore indicate a gradual transition from site-dominated adsorption toward cooperative water-network stabilization.

**Figure 6:**
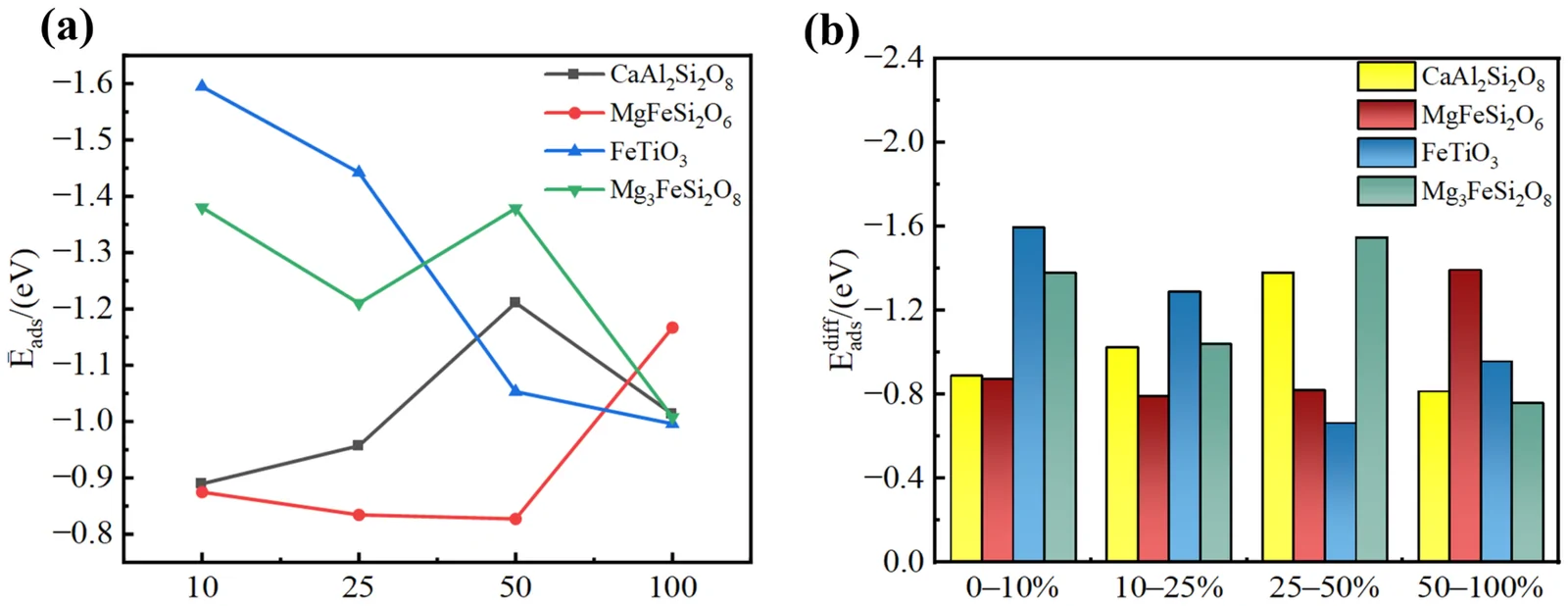
Coverage-dependent adsorption energies of multiple H_2_O molecules on representative lunar mineral surfaces: (**a**) average adsorption energy and (**b**) differential adsorption energy.

The average adsorption energies in Figure 6a provide a quantitative measure of the overall stability of adsorbed H_2_O molecules at different coverages. At 10% coverage, FeTiO_3_ (100) exhibits the strongest average adsorption energy of −1.59 eV, followed by Mg_3_FeSi_2_O_8_ (010) with −1.38 eV. In contrast, CaAl_2_Si_2_O_8_ (001) and MgFeSi_2_O_6_ (110) show weaker average adsorption energies of −0.89 and −0.87 eV, respectively. This trend is consistent with the single-water adsorption results, confirming that Ti- and Fe-containing surfaces provide stronger initial anchoring sites for the first adsorbed water molecules.

The differential adsorption energies in Figure 6b provide further evidence for this coverage-dependent transition. The highly negative *E_diff_* values of FeTiO_3_ and Mg_3_FeSi_2_O_8_ at 10% coverage correspond to the initial capture of H_2_O by strong Ti/Fe-related anchoring sites. With increasing coverage, the weakening or recovery of *E_diff_* reflects the competition between the occupation of weaker surface sites and the cooperative stabilization provided by hydrogen bonding. In particular, the more negative *E_diff_* of MgFeSi_2_O_6_ at 100% coverage indicates that a connected hydrogen-bonded water layer can compensate for its weaker initial anchoring ability. Therefore, the high-coverage adsorption behavior is not merely a dilution of metal-site binding strength, but also reflects the emergence of network-assisted stabilization.

With increasing coverage, the *E_avg_* values do not vary monotonically, indicating that high-coverage adsorption cannot be explained simply by the strength of the initial metal anchoring sites. For FeTiO_3_ (100), *E_avg_* gradually becomes less negative from −1.59 eV at 10% coverage to −1.00 eV at 100% coverage. This trend indicates that Ti-related anchoring dominates the initial adsorption stage, whereas the average contribution of direct metal-site coordination decreases as additional water molecules occupy less favorable local environments and participate in intermolecular interactions. This progressive weakening is primarily attributed to the saturation of the strongest Ti sites: once these optimal anchors are occupied, subsequent water molecules are forced to adsorb at intrinsically weaker surface positions. A similar weakening at full coverage is observed for Mg_3_FeSi_2_O_8_ (010), where *E_avg_* decreases from −1.38 eV at 10% to −1.21 eV at 25%, recovers to −1.38 eV at 50%, and then becomes less negative at 100% coverage (−1.01 eV). This nonmonotonic behavior suggests that intermediate coverage can provide a favorable balance between surface anchoring and cooperative water-water stabilization, whereas excessive coverage may introduce geometric crowding and reduce the average stabilization per molecule.

By contrast, CaAl_2_Si_2_O_8_ (001) and MgFeSi_2_O_6_ (110) show different coverage responses. For CaAl_2_Si_2_O_8_ (001), *E_avg_* becomes progressively more negative from −0.89 eV at 10% to −1.21 eV at 50%, indicating that the accumulation of additional H_2_O molecules enhances the collective stability of the adsorbed layer at intermediate coverage. This enhancement arises from the growing H-bond network among closely spaced water molecules, which provides cooperative stabilization that partially compensates for the occupancy of weaker adsorption sites. However, *E_avg_* becomes less negative again at 100% coverage (−1.01 eV), suggesting that the stabilizing effect of further water addition weakens after the development of a denser adsorbed layer. For MgFeSi_2_O_6_ (110), *E_avg_* remains relatively weak from 10% to 50% coverage, but becomes significantly more negative at 100% coverage (−1.16 eV). This indicates that although the initial adsorption ability of MgFeSi_2_O_6_ is relatively limited, high-coverage water-water interactions can enhance the stability of the adsorbed water layer.

The differential adsorption energies in Figure 6b further clarify the stepwise adsorption mechanism. All *E_diff_* values are negative, indicating that additional H_2_O adsorption is energetically favorable over the investigated coverage range. In the initial 0–10% interval, FeTiO_3_ (100) and Mg_3_FeSi_2_O_8_ (010) show the most negative *E_diff_* values of −1.59 and −1.38 eV, respectively, confirming that their exposed transition-metal sites provide the strongest driving force for initial water capture. In comparison, the *E_diff_* values of CaAl_2_Si_2_O_8_ (001) and MgFeSi_2_O_6_ (110) are less negative, both close to −0.88 eV, reflecting their weaker initial anchoring ability.

In the intermediate coverage region, the *E_diff_* values reveal how site saturation and cooperative stabilization compete with each other. For FeTiO_3_ (100), *E_diff_* becomes less negative from −1.29 eV in the 10–25% interval to −0.66 eV in the 25–50% interval. This indicates that once the strongest Ti-related sites are partially occupied, the newly added H_2_O molecules gain less stabilization from direct water-mineral coordination. In contrast, CaAl_2_Si_2_O_8_ (001) and Mg_3_FeSi_2_O_8_ (010) show strongly negative *E_diff_* values in the 25–50% interval, reaching −1.38 and −1.55 eV, respectively. Combined with the more laterally connected configurations in Figure 5, this suggests that the newly introduced H_2_O molecules in this coverage interval are effectively stabilized by local cooperative interactions, including surface anchoring, water orientation adjustment, and hydrogen-bond-assisted water-water interactions.

At the highest coverage interval of 50–100%, the differential adsorption behavior becomes strongly mineral-dependent. MgFeSi_2_O_6_ (110) exhibits a markedly more negative *E_diff_* value of −1.39 eV, which is consistent with its pronounced decrease in *E_avg_* at 100% coverage. This indicates that once sufficient water molecules are present on the MgFeSi_2_O_6_ surface, the adsorbed layer can be further stabilized by the development of a more connected water-water interaction network. Unlike FeTiO_3_ or Mg_3_FeSi_2_O_8_, where geometric crowding eventually offsets the H-bond gain at full coverage, the atomic topology of MgFeSi_2_O_6_ (110) appears to provide a particularly favorable geometric matching for extended H-bond connectivity, allowing the cooperative network effect to dominate overcrowding-induced losses. In contrast, Mg_3_FeSi_2_O_8_ (010) and CaAl_2_Si_2_O_8_ (001) show less negative *E_diff_* values of −0.76 and −0.82 eV in the same interval, suggesting that the incremental stabilization from additional water molecules becomes weaker after the intermediate-coverage stabilization stage. For FeTiO_3_ (100), *E_diff_* becomes more negative again in the 50–100% interval (−0.96 eV) compared with the 25–50% interval, implying that water-water interactions partly compensate for the weakening of direct anchoring at high coverage.

Overall, the coverage-dependent adsorption results reveal that water accumulation on lunar mineral surfaces is not a simple linear extension of single-water adsorption. At low coverage, adsorption is mainly governed by the intrinsic strength of exposed metal sites. At intermediate coverage, strong-site occupation, molecular orientation, and local water-water interactions jointly determine the adsorption stability. At high coverage, adsorbed H_2_O molecules tend to extend laterally along the surface and form a more connected hydrogen-bond-assisted network, which becomes an important contributor to the collective stabilization of the adsorbed layer. These results demonstrate that mineral-dependent water retention is controlled by the coupled effects of initial metal-site anchoring and coverage-induced cooperative water-water interactions.

## 4. Conclusions

In this work, first-principles calculations were performed to systematically investigate single- and multi-water adsorption on representative surfaces of four major lunar regolith minerals: anorthite (CaAl_2_Si_2_O_8_), Fe-bearing pyroxene (MgFeSi_2_O_6_), ilmenite (FeTiO_3_), and Fe-bearing olivine (Mg_3_FeSi_2_O_8_). Moving beyond a mere comparison of adsorption energies, this study establishes a unified theoretical framework that links mineral surface chemistry to coverage-dependent water retention mechanisms. Single-water adsorption reveals that H_2_O preferentially anchors at exposed metal sites via O-M coordination, with Ti and Fe sites exhibiting stronger initial binding than Mg, Ca, or Al sites. The Hard–Soft Acid–Base (HSAB) principle qualitatively rationalizes this low-coverage site preference based on Lewis acidity, where the accessible d-orbitals of Ti/Fe centers enable pronounced orbital-mediated interfacial bonding and polarization that extend beyond simple electrostatic matching. However, this HSAB-driven selectivity is primarily confined to the isolated or low-coverage regime. Crucially, water accumulation is not a simple linear extension of single-site affinity. As coverage increases, the stabilization mechanism undergoes a fundamental transition. At low coverage, adsorption is localized and site-specific, dictated by cation acidity. At high coverage, the adsorption behavior is no longer governed by isolated cation acidity but by the cooperative formation of laterally connected hydrogen-bond networks. The differential adsorption energies demonstrate that surface topology and geometric compatibility for hydrogen-bond connectivity become the decisive factors for retaining a dense water layer. Based on the above results, the water adsorption and accumulation on lunar mineral surfaces can be summarized as a dual-stage cooperative mechanism. In Stage I (low coverage, <~25%), adsorption is dominated by exposed Lewis acidic sites. Water molecules preferentially anchor at Ti, Fe, and Ca sites via O-M coordination, where the accessible d-orbitals and stronger polarizability of Ti/Fe centers enable pronounced orbital-mediated interfacial bonding that extends beyond simple electrostatic matching. In Stage II (high coverage, >~50%), once the strongest sites become saturated, additional water molecules are no longer stabilized primarily by direct metal coordination. Instead, their stability is governed by the geometric ability of the mineral surface to support lateral hydrogen-bond networks (H_2_O···H_2_O and H_2_O···surface-O). In this regime, the surface acts as a structural template, while the initial electronic preference of cations takes a secondary role. We therefore propose a dual-stage cooperative mechanism for water adsorption on lunar mineral surfaces: it is initiated by cation-specific Lewis acid–base anchoring at low coverage and ultimately sustained by coverage-driven, topology-dependent hydrogen-bond network stabilization at high coverage. These findings suggest that predictive models for volatile retention on airless bodies must account for both the electronic activity of surface cations and the structural topology of mineral surfaces.

## Figures and Tables

**Table 1:** Calculated surface energies of the candidate low-index surfaces.

Mineral	Surface	Surface Energy/eV A^−2^	Surface Energy/J m^−2^
CaAl_2_Si_2_O_8_	(001)	0.03952	0.633
CaAl_2_Si_2_O_8_	(010)	0.03549	0.569
MgFeSi_2_O_6_	(010)	0.01747	0.280
MgFeSi_2_O_6_	(110)	0.03846	0.616
FeTiO_3_	(001)	0.03384	0.542
FeTiO_3_	(100)	0.03581	0.574
Mg_3_FeSi_2_O_8_	(001)	0.05083	0.814
Mg_3_FeSi_2_O_8_	(010)	0.08030	1.287

**Table 2:** Calculated adsorption energies, M-O distances, and ICOHP values for single-H_2_O adsorption on the representative mineral surfaces.

Mineral Surface	Site	E_ads_ (eV)	M-O Distance (Å)	ICOHP (eV)
CaAl_2_Si_2_O_8_ (001)	Ca	−1.05	2.321	−0.529
	Al	−0.76	3.246	−0.096
MgFeSi_2_O_6_ (110)	Fe	−0.73	2.117	−1.590
	Mg	−0.56	2.178	−1.110
FeTiO_3_ (100)	Fe	−0.33	2.137	−1.250
	Ti	−1.29	2.248	−1.600
Mg_3_FeSi_2_O_8_ (010)	Fe	−1.22	2.135	−0.990
	Mg	−0.71	2.011	−0.310

## Data Availability

The original contributions presented in this study are included in the article. Further inquiries can be directed to the corresponding authors.
